# *In Vitro* Growth of Thymic Tumor Cell Lines from *Xenopus*

**DOI:** 10.1155/1992/41823

**Published:** 1992

**Authors:** Louis du Pasquier, Jacques Robert

**Affiliations:** Basel Institute for Immunology Grenzacherstrasse 487 Postfach Basel 4005 Switzerland

**Keywords:** Xenopus, lymphoid tumor, cell lines

## Abstract

A spontaneous lymphoid thymus tumor was discovered in a male *Xenopus* of the MHC *ff* genotype. The tumor cell can be transplanted in histocompatible larval *ff* hosts, but not in *ff* adults unless irradiated (3000 rad). The tumor is rejected by allogeneic hosts. The tumor cells express neither markers of the B-cell lineage nor MHC encoded molecules; they express only markers of the T-cell lineage. Its lymphoid population is clonal as revealed by the existence of a stable rearrangement pattern of the immunoglobulin genes. Cell lines growing continuously *in vitro* have been derived from the tumor.


*Developmental Immunology*, 1992, Vol. 2, pp. 295–307
Reprints available directly from the publisher
Photocopying permitted by license only

© 1992 Harwood Academic Publishers GmbH
Printed in the United Kingdom

### *In Vitro* Growth of Thymic Tumor Cell Lines from *Xenopus*

LOUIS DU PASQUIER and JACQUES ROBERT

*Basel Institute for Immunology, Grenzacherstrasse 487, Postfach, 4005 Basel, Switzerland*

A spontaneous lymphoid thymus tumor was discovered in a male *Xenopus* of the MHC *ff* genotype. The tumor cell can be transplanted in histocompatible larval *ff* hosts, but not in *ff* adults unless irradiated (3000 rad). The tumor is rejected by allogeneic hosts. The tumor cells express neither markers of the B-cell lineage nor MHC encoded molecules; they express only markers of the T-cell lineage. Its lymphoid population is clonal as revealed by the existence of a stable rearrangement pattern of the immunoglobulin genes. Cell lines growing continuously *in vitro* have been derived from the tumor.

KEYWORDS: Xenopus, lymphoid tumor, cell lines.

### INTRODUCTION

Lymphoid tumors and tumor-derived lymphoid cell lines have been major tools for understanding the mechanisms underlying B- and T-cell differentiation in mammals. Because such systems were known until now only in warm-blooded vertebrates, these tools were not available for other interesting, cold-blooded vertebrate models. In amphibians, one of the most widely used models outside mammals because of their phylogenetic position, and because of their ontogenetic peculiarities, there has been only a few reports of lymphoid tumors, and none of them could be used as a source of cell lines. In urodeles, a lymphosarcoma transplantable only between histocompatible strains was reported in the Axolotl *Ambystoma mexicanum* (De Lanney et al., 1964; De Lanney and Blackler, 1969). Another putative lymphosarcoma was described in *Triturus pyrrhogaster*, but it turned out to be caused by an acid-fast bacterium and was likely an infectious granuloma (Inoue et al., 1965). In anurans, two lymphoid tumors have been mentioned in the leopard frog, *Rana pipiens*. One was an undefined lymphosarcoma (Duryee, 1969); the other was a plasmacytoma (Schochet and Lampert, 1969) that developed 2.5 months after transplantation of a Lucke renal carcinoma. In *Xenopus*, the anuran model most widely used by immunologists, what was thought to be a malignant lymphosarcoma (Balls, 1962) found primarily in the liver, spleen, and kidney, and transmissible by cell-free extracts, even to hosts of different zoological orders (Balls and Ruben, 1967, 1968; Hadji-Azimi, 1970a, 1970b) turned out to be an infectious granuloma (Asfari, 1988; Asfari and Thiébaud, 1988). A spontaneous lymphoblastic lymphoma was reported in *Xenopus*, and none of its histological aspects are compatible with a malignant origin (quoted in Asfari, 1988). A transplantable lymphoma was induced in *Xenopus* by exposure to N-methyl-nitrosourea, but it was not fully characterized nor adapted to grow indefinitely *in vitro* (Balls et al., 1981, 1983). It was therefore an important event when we detected in our colony one animal carrying a tumor in the thymus. We report here for the first time the description of a spontaneous lymphoid tumor in *Xenopus* and how stable cell lines growing continuously *in vitro* have been derived from it.

### RESULTS

### Occurrence of a Spontaneous Lymphoid Tumor in the Thymus

On December 7, 1990, Roland Jost, the caretaker of the frog colony at Basel, brought in a *Xenopus* male of the MHC homozygous *ff* strain, born in 1986, with an outgrowth in the region of the right thymus. The animal was anesthetized, and about half of what appeared to be a thymus tumor was dissected out and kept on ice in L15 medium. The animal was sewn up and returned to the aquarium. The tumor was cut into pieces of 1 to 2 mm and immediately transferred subcutaneously to the head region of six young postmetamorphic *ff* animals. The rest of the tumor was kept for histology. A cell suspension stained with Giemsa after cytocentrifugation showed cells of lymphoblastoid aspect. Another cell suspension was prepared, washed, and injected intraperitoneally into tadpoles ($2\times10^5$ cells/inoculum); the remainder of the suspension was put into culture in 24-well Costar trays at about $1\times10^6$ cells/ml, and kept at 27°C in a humidified 3% $CO_2$ incubator in various culture media (see the next section).

1. The tumor is transplantable in isogenic *ff* animals, but not in allogenic hosts. Tumor cells also grew when injected into *ff* tadpoles. Upon injection of 200,000 cells into a tadpole, about $6\times 10^6$ cells are recoverd 3 weeks later. This technique allowed us to maintain large numbers of tumor cells, especially when injections were done in sodium perchlorate blocked tadpoles that do not metamorphose (Pflugfelder, 1961). Withdrawal of tumor cells can be performed every week with a glass pipette, as described for peritoneal fluid (Du Pasquier et al, 1985). Two attempts were made to grow tumor cells in fully allogeneic tadpoles of the LG15 clone with the MHC haplotype *ac*; no tumor growth was obtained whether or not the animals were thymectomized. All cells of tumor origin were gone after 3 weeks, and no more could be rescued when cultures were attempted from the peritoneal fluid of these injected allogeneic animals (Table 1).

2. Transplanted fragments developed in young postmetamorphic histocompatible animals, and injected cells introduced subcutaneously could also generate tumors. Tumor fragments grew locally, but metastases were frequent. New tumors like the original developed chiefly in the thymus (Fig. 1), but also in the spleen, liver, and in the abdominal or dorsal musculature near the site of the injection. Occasional metastases were observed in the skin, including that of the original tumor bearer. Tumors did not develop in fully grown histocompatible *ff* adults, even after injection of large numbers of tumor cells ($4\times 10^6$/animal), unless whole-body irradiation was given. In this case, solid tumors of 2 grams could be obtained 4 months later.

3. Phenotype of the tumor (Figs. 3 and 4). Frozen sections of the original tumor and of subsequently grown metastases were stained with

TABLE 1
Results of Transplantation Experiments with Tumor-Tissue Fragments and Tumor Cells

| Nature of transplant | Nature of host | No. of animals | Results |
|---|---|---|---|
| Solid tissue 3 mm$^3$ | *ff* 1 month postmetamorphosis | 6 | Local growth and metastasis in host thymus, liver, spleen and skin within 1–2 months |
| | *ff* adult 4 years old | 6 | No growth |
| | *ff* adult 4 years old (3000 rad) | 4 | Metastasis in liver and spleen 4 months later |
| Cell intraperitoneally ($2\times10^5$) | *ff* 1 month postmetamorphosis | 40 | Ascites and metastasis in thymus, spleen, liver and at the site of injection within 1 month |
| | *ff* adult 4 years old | 4 | No growth |
| ($2\times10^6$) | *ff* adult 4 years old | 4 | No growth |
| | *ff* adult 4 years old (3000 rad) | 43 | Metastasis in liver and spleen |
| Cell intraperitoneally ($2\times10^5$) | *ff* tadpoles stages 54–56 | 10 | Ascites, circa $2\times10^7$ cells recovered 1 week later/animal. Postmetamorphic animals also had ascites and metastasis. |
| | *ff* tadpoles blocked with sodium perchlorate | 100 | Ascites |
| | ac(LG15) MHC disparate tadpoles | 12 | No growth |
| | ac(LG15) MHC tadpoles thymectomized at day 20 | 12 | No growth |
| Supernatant of tumor cell line (50 $\mu$l) | *ff* tadpoles blocked with sodium perchlorate | 6 | No growth |

various monoclonal antibodies in indirect immunofluorescence assays. In parallel, single cell suspensions were prepared and analyzed by flow cytometry. Examples of sections are presented in Figs. 2 and 3; flow cytometric profiles are given in Fig. 4; results are summarized in Table 2. Whether the cells were taken from the original thymic tumor or from metastases in the spleen, the patterns of staining were identical. Although the tumor tissue is not stained at all, many plasma cells surrounding the nodule of tumor tissue are stained by anti-Ig reagent (Fig. 3). Anti-MHC class I monoclonal antibodies gave the same result as with larval tissue; an intracellular and intranuclear staining with TB17 and TB1, respectively (Fig. 3), but no stain on the surface (Flajnik et al 1990b) (Table 2). Anti-MHC class II (14A2 and AM20) did not stain the tumor cells (Figs. 3 and 4, Table 2).

In summary, the tumor does not express, neither in its cytoplasm nor on its membrane, any marker of the B-cell lineage (Ig and MHC class II) found in normal B cells. The only monoclonal antibodies that reacted were the ones recognizing determinants generally distributed on lymphocyte or hematopoietic tissue (AM14, RC47, and f1F6), or T-cell specific determinants (the 120-kd specific epitope recognized by X21.2 and the CD8 *Xenopus* equivalent antigenic determinant picked up by AM22). Some single-cell staining patterns were not homogenous. When tested on *in vivo* grown tumor cells, anticlass I and II monoclonal antibodies stained a fraction of the cells, but we attribute their staining to contaminating host-derived cells in view of the homogeneity of phenotypes found later on the cell lines (see further section and Fig. 4). The tumor cells expressed more of the two T-cell specific epitopes X21.2 and AM22 than the normal T cell, as can be judged from the flow cytometric profile (Fig. 4).

4. Clonal nature of the tumor. DNA was prepared from tumor cells digested with several enzymes, and Southern blots were hybridized with probes specific for Ig $J_H$ (Schwager et al., 1988), $V_H7$ (Haire et al., 1990), or MHC $\alpha 3$ (Flajnik et al., 1990a). Because the band corresponding to the unrearranged $J_H$ was no longer visible in the tumor; the IgH locus seemed to be rearranged on both alleles. The same bands, that is, the same rearrangements, were found in all

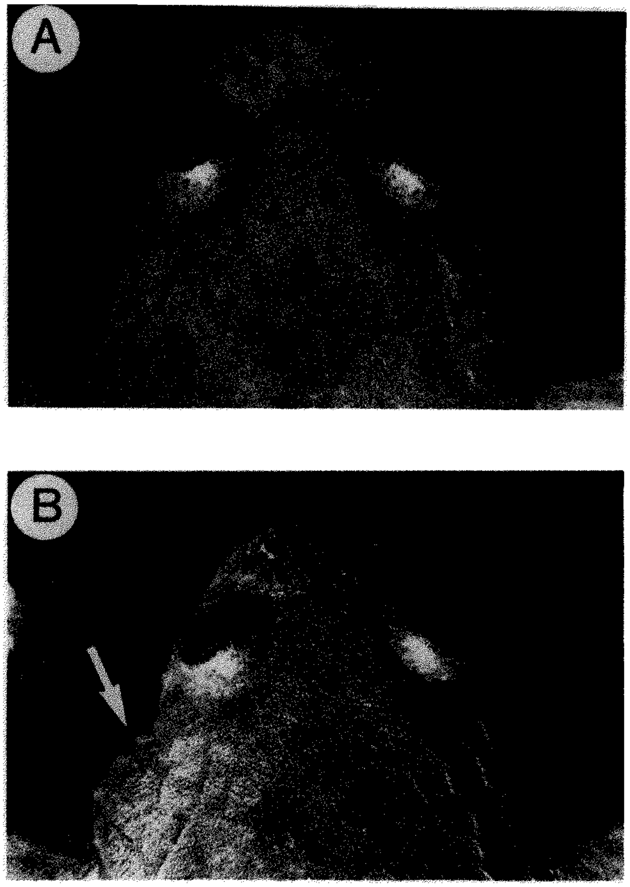

FIGURE 1. Aspect of *Xenopus* with a thymus tumor. (A) Control animal, *ff* strain, 2 months after metamorphosis. (B) Experimental animal, *ff* strain, 1 month after metamorphosis injected intraperitoneally with $2 \times 10^5$ tumor cells. Arrow points to the tumor in the thymus.

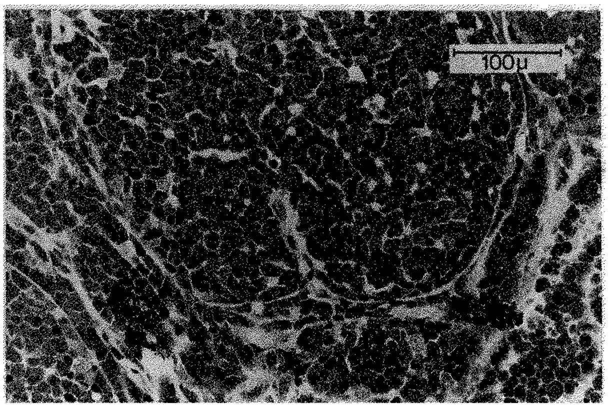

FIGURE 2. Section through the original thymic tumor, showing infiltration of the host thymic tissue by the lymphocytes of the tumor. The pigment cells correspond to the region originally separating the cortex from the medulla (Notch's stain, semithin section).

subsequent DNA preparations made from the tumor grown *in vivo* over the following 8 months (one sample analyzed every month) (Fig. 5). The lymphoid cell population of the tumor seems to be clonal.

The tumor-cell DNA was compared with nor-

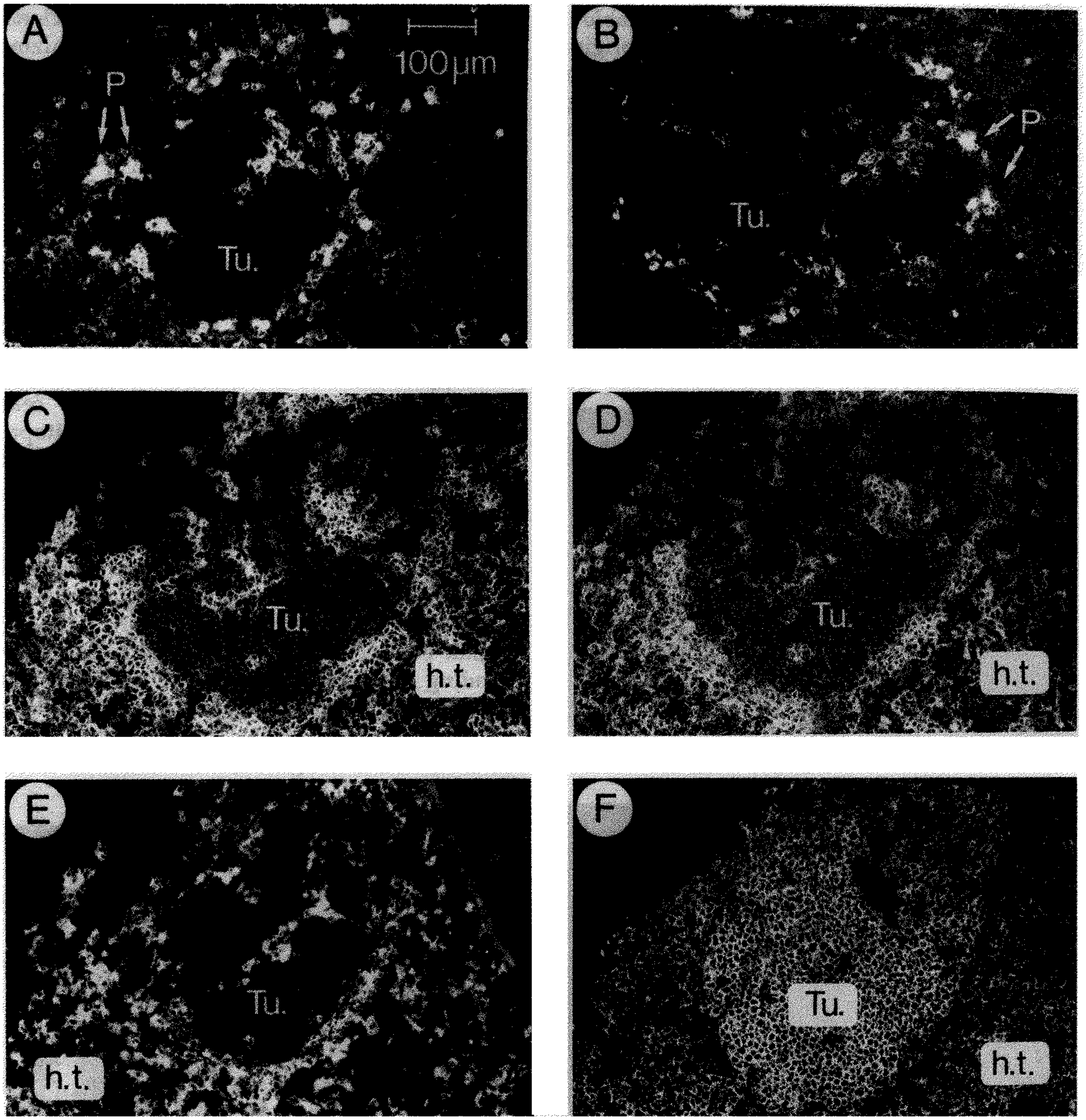

FIGURE 3. Immunohistology on tumor sections. (A) Anti-IgM staining (mAb 10A9) on the original thymic tumor tissue. Tu.: tumor tissue nodule. P: Thymic plasma cell. (B) As (A) but on a section from a spleen metastasis obtained by injecting one postmetamorphic animal with $2\times10^5$ tumor cells. Tu.: Tumor tissue nodule. P: Splenic plasma cell. (C) Anti-MHC class I (mAb TB17) on the original thymic tumor tissue. The nodule of tumor tissue (Tu.) stains weakly, whereas the host tissue (h.t.) is brightly stained. (D) Anti-MHC class I (mAb TB1) contiguous section. Typical intracellular and intranuclear stain. mAbs TB17 and TB1 both fail to stain the cell surface. (E) Anti-MHC class II (mAb AM20). The thymic tumor nodule (Tu.) does not stain, whereas the surrounding host thymic tissues are positive (h.t.). (F) Anti-CD8 (mAb AM22). All lymphocytes from the tumor nodule are brightly stained.

mal *ff* red-cell DNA and also with the DNA of the leg of the original tumor-bearing animal, which had been preserved by freezing, the rearrangements are weakly visible. Then the tumor cells must have colonized the leg or have been circulating through the animal. Indeed, shortly before its death, the original animal showed metastases in the skin, out of which cells were isolated and injected into *ff* tadpoles, where they proliferated.

### Tumor-Derived Stable Cell Lines

1. Cells of the original tumor were kept *in vitro* in various culture media, all derived from the Leibowitz L15 base media in which T-cell proliferation and antibody production can be obtained *in vitro* (Weiss and Du Pasquier, 1973). One of the media contained 20% medium conditioned by *Xenopus* kidney cell line A6 (Rafferty, 1969) growing to confluence. In this medium, proliferation of tumor cells continued for several days, but lymphocytes were soon overgrown by other cell types of host origin (polymorphonuclear cells, granulocytes, esoinophils, pigment cells), which seemed to respond to some of the factors brought in by the A6 cell line supernatant (see Paragraph 2 and Fig. 6). Such mixtures were then injected back into blocked *ff* tadpoles, where lymphoid proliferation was dominant. Cells were then withdrawn 3 weeks later from the peritoneal cavity and cultured again *in vitro.* After four passages of this type, in May 1991, in 1 well out of 24 of a culture tray seeded with such cells, an accumulation of proliferating nonadherent large lymphocytes was detected, and these cells quickly overtook all the other cell populations in the well. After their culture was split two times, a pure lymphoid cell line was established (Figs. 4 and 6). This line had the same Ig rearrangement as the tumor, and the same phenotype in flow

TABLE 2
Staining of Tumor, and Tumor Cell Line with Monoclonal Antibodies

| Monoclonal antibodies | | References | Specificity | Results | | |
|---|---|---|---|---|---|---|
| | | | | Tumor | | Cell line |
| | | | | Tissue sections | Single cells Flow cytometry | Single cells Flow cytometry |
| Anti-Ig | 10A9 | 1 | IgM | – | ND | ND |
| | 11D5 | 1 | IgY | – | ND | ND |
| | 410D9 | 2 | IgY | – | ND | ND |
| | 3B2 | 3 | IgL1 | – | – | – |
| | 1E9 | 3 | IgL2 | – | – | – |
| | 409B8 | 2 | IgL3 | – | – | – |
| | 10C10 | 1 | $IgV_Ha$ | – | – | – |
| | 14B4 | 1 | $IgV_Hb$ | – | – | – |
| Anti-MHC | TB1 | 4 | Class I | +(nuclei) | – | – |
| | TB17 | 4 | Class I | +(cytopl.) | – | – |
| | AM20 | 5 | Class II | – | – | – |
| | 14A2 | 5 | Class II | – | – | – |
| Anti-T cell | AM22 | 6 | CD8 | + | + | + |
| | X21.2 | 7 | T cell | + | + | + |
| Anti-lymphocytes and other markers | AM14 | 8 | Lymphocytes | + | + | + |
| | F1F6 | 9 | Leucocytes Erythrocytes | + | + | + |
| | RC47 | 6 | Leucocytes | + | + | + |

1. Hsu and Du Pasquier 1984
2. Hsu and Du Pasquier, unpublished
3. Hsu et al., 1985, 1991
4. Flajnik et al., 1991
5. Flajnik et al., 1990b
6. Du Pasquier and Flajnik 1991
7. Nagata 1985
8. Flajnik, unpublished
9. Flajnik et al., 1988

cytometry studies (Table 2). It was adpated to normal medium (i.e., the same composition as mentioned before but without A6 supernatant) containing 5% FCS. The cells can also grow in medium not supplemented with IMDEM, but we prefer to use the full "rich" mixture, which gives a slightly better yield. The original line and its subclones are now grown in a Nunc cells factory to produce up to $10^8$ cells/week. We have not been able to achieve concentrations greater than $1\times10^6$/ml.

The line was cloned, and two categories of subclones were isolated: fast-growing (24-h generation time at 27°C) and slow-growing (38 h). Two rapidly growing clones, B3D3 and B3B7, which seem to be able to grow at a higher cell concentration, are now used preferentially. The supernatant of these tumor-cell cultures did not induce new tumor growth *in vivo* (six out of six negative, 8 months after injection, Table 1).

2. As mentioned before, in the initial phase of the *in vitro* adaptation, the survival of the cells depended on the presence of growth factors present in the supernatant of the A6 kidney cell line. During this phase, many nontumorous cell types grew. This prompted us to analyze further the effect of these factors on normal tissues, since it is of potential interest to be able to grow cells of various lineages in *Xenopus*. Thymus, spleen fragments, and peritoneal cells from tadpoles were set up in culture under the same conditions as the tumor in L15 medium+20% A6.

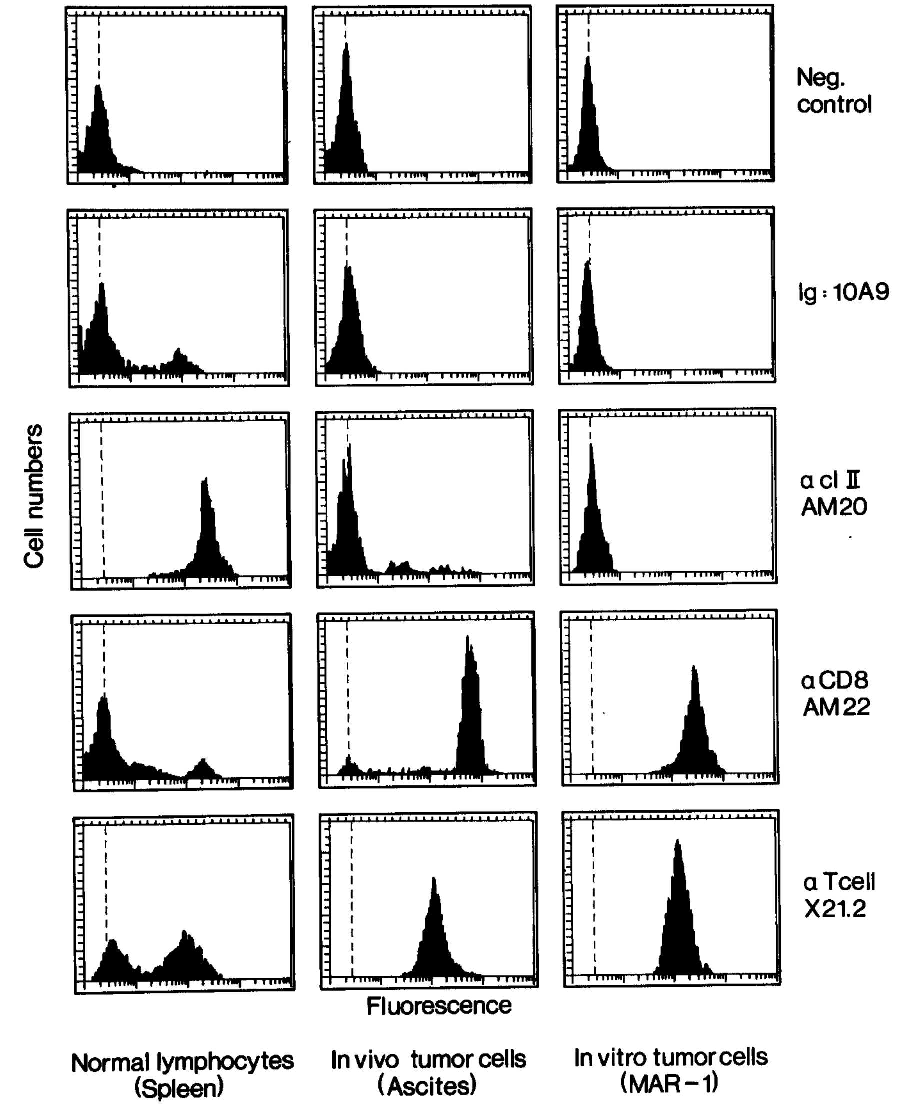

FIGURE 4. Flow cytometric analysis of tumor cells versus normal lymphocytes. The specificity of the monoclonal antibodies is given in Table 2.

Presence of A6 supernatants markedly improved the survival of cells in all cultures. In parallel control-tissue culture with L15 normal medium, approximately 50% of cells are already dead after 3 days, and less than 10% remain alive after 1 week. In A6 medium, the cells rapidly proliferate. Fibroblasts generally grew in the first 3 days, covering the well with a thin monolayer; in a few cases, melanocytes also proliferated. Lymphoid cells usually did not proliferate during this period, but they showed signs of activity (active membrane movement) and the number of blast cells increased rapidly. After 1 week, there is significant proliferation in some wells and not in others. Some of these cell cultures were maintained and amplified for more than 1 month. There was also a great heterogeneity in cell-type composition among wells. In cytospin preparations stained with Giemsa, one can see, in addition to lymphocytes, the various members from the granulocyte family including monocytes, neutrophils, eosinophils, and polymorphonuclear cells. Their relative proportion is quite different from one well to another. In some samples, eosinophils were highly overrepresented (more than 10%), and in others, they were almost absent (less than 0.2%) (Fig. 6). The cell type that dominated in all cultures is the polymorphonuclear leucocyte. In one case, we were able to propagate such cells in 75 $cm^2$ flasks, but unfortunately this culture was lost by contamination. Lymphocytes are still present in culture after 1 month, but they seem not to proliferate. B cells disappear preferentially in the early phase of the culture.

### Karyotypes of the Tumor and of the Cell Line

Chromosome spreads were prepared from Colcemid blocked tumor lymphocytes, as previously

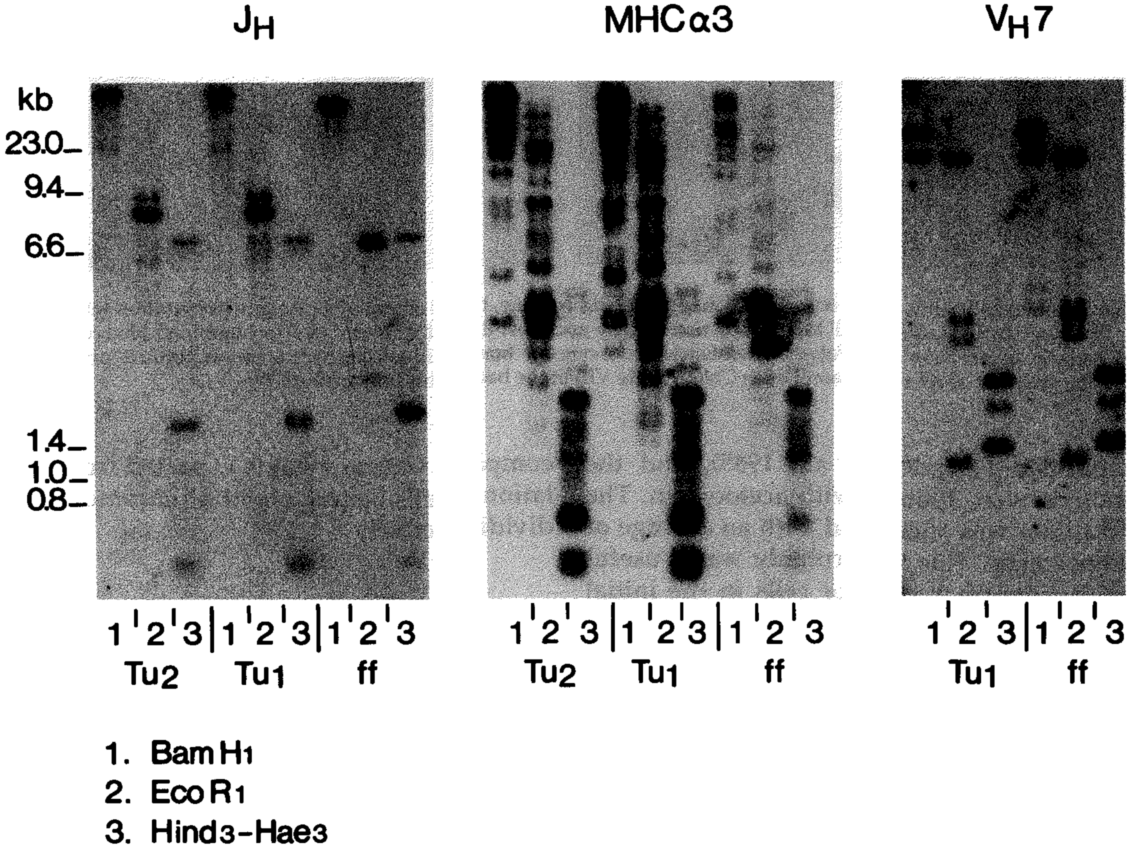

FIGURE 5. Southern blot of tumor DNA. Hybridization patterns of BamHI, EcoRI, and HindIII/HaeIII digests of two different samples of the tumor, $Tu_1$ and $Tu_2$, taken at 2-month intervals from cells grown *in vivo* and compared to red-cell DNA of the *ff* host cells. The $J_H$ probe shows the rearrangement of Ig genes on the two alleles. Trace amounts of germ-line configuration are still visible because of contamination with host peritoneal cells. The MHC and $V_H$ probes show the identity of the hybridization patterns between the tumor samples and the germ-line red-cell DNA sample.

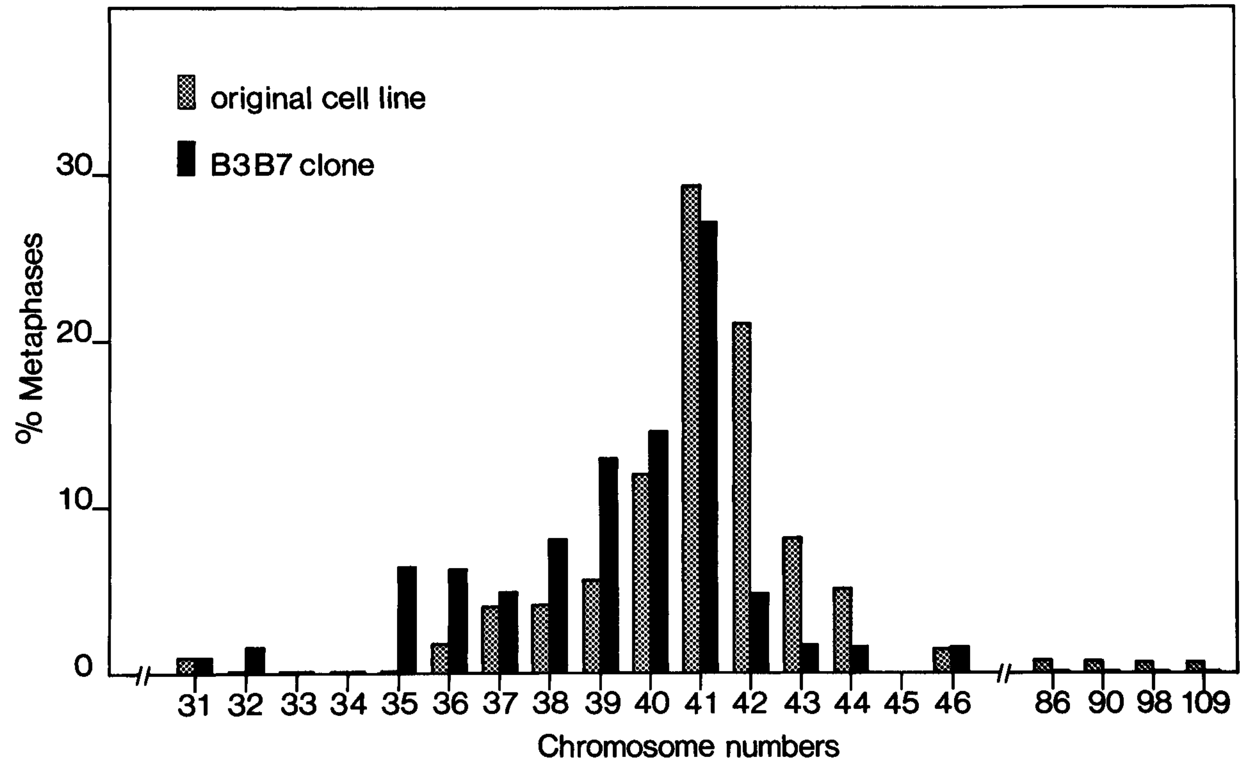

FIGURE 7. Distribution of chromosome numbers in the tumor cell line.

and transplanted into *ff* histocompatible animals in order to obtain a large quantity of material should make it an interesting tool for several aspects of *Xenopus* biology and immunology. These cells are nonadherent and hence easy to recover, clone, and use in flow cytometry. It could easily be experimentally the most facile *Xenopus* cell line available.

### Several Developments Can Be Envisaged

(1) The cell line could be manipulated in order to obtain fusion partners offering possibility of selection. We are now trying to introduce neomycin-resistant gene; attempts to make HAT-sensitive azaguanine resistant mutants have so far failed. The line could also be used in transient transfection assays. (2) It is an abundant source of at least two molecules of immunological interest, CD8 and the T-cell specific marker X21.2, for which enough material can be purified for protein sequencing. (3) The cell line has an apparent larval phenotype; like larval cells, it does not express class I molecules (Flajnik et al., 1986; Du Pasquier et al., 1979). If confirmed, this could also be exploited in differential screening experiments aimed at distinguishing differential gene expressions during the ontogeny of the *Xenopus*. (4) Although we do not know the origin of the tumor, whether it arose because of transmission by a virus or translocation of oncogenes, this tumor could be helpful for studying certain aspects of lymphocyte differentiation. In this respect, it will be essential to analyze further and confirm the apparent B–T hybrid nature of the tumor in which some Ig genes are rearranged and T-cell markers are expressed, even up-regulated. Because the cell lines and the tumor exhibit several characteristics of T cells, it will be interesting to investigate putative T-cell functions and look for other T-cell markers, whenever reagents are available. Monoclonal antibodies should all be raised against the tumor. If certain epitopes like the previously mentioned T-cell markers are up-regulated, the cell lines constitute a better source of antigen than the normal T cells. On the other hand, the presence of two rearrangements within the Ig locus demands that we investigate

B-cell parameters with other techniques. The sequence of the rearrangements will tell us whether we are dealing with DJ or VDJ rearrangement, and it may become necessary to analyze mRNA for transcription of all the genes for which no product has been detected. The line might also harbor rearrangements at the T-cell receptor locus and be a convenient source of cloned material for further studies.

The fact that the tumor did not grow in nonirradiated, fully grown histocompatible sibs, nor in thymectomized allogeneic adult hosts is somewhat surprising from an immunological point of view. The *ff* adult animals used for these assays accept skin grafts for at least 200 days; therefore, we anticipated at least an initial growth of the tumor. However, whatever the route of injection (intravenous and subcutaneous injections were also tried), the size of the inoculum, or the inoculation schedule, no growth was ever recorded. Maybe the tumor cells express a potent non-MHC antigen that would not be present in the skin. Thymic tumors, similar to the one originally detected, were obtained only when young postmetamorphosis animals were used. Since young, postmetamorphosis animals accept the tumor transplant and adults do not, there must be a period when immunity develops. Future experiments will be done to find out whether this period coincides with the acquisition of the full immunological maturity of the host (Du Pasquier and Chardonnens, 1975) or whether the apparent resistance is due to a homing problem.

Of practical importance is the finding that the supernatant of the A6 kidney cell line support the growth of several cell types of the hemato-

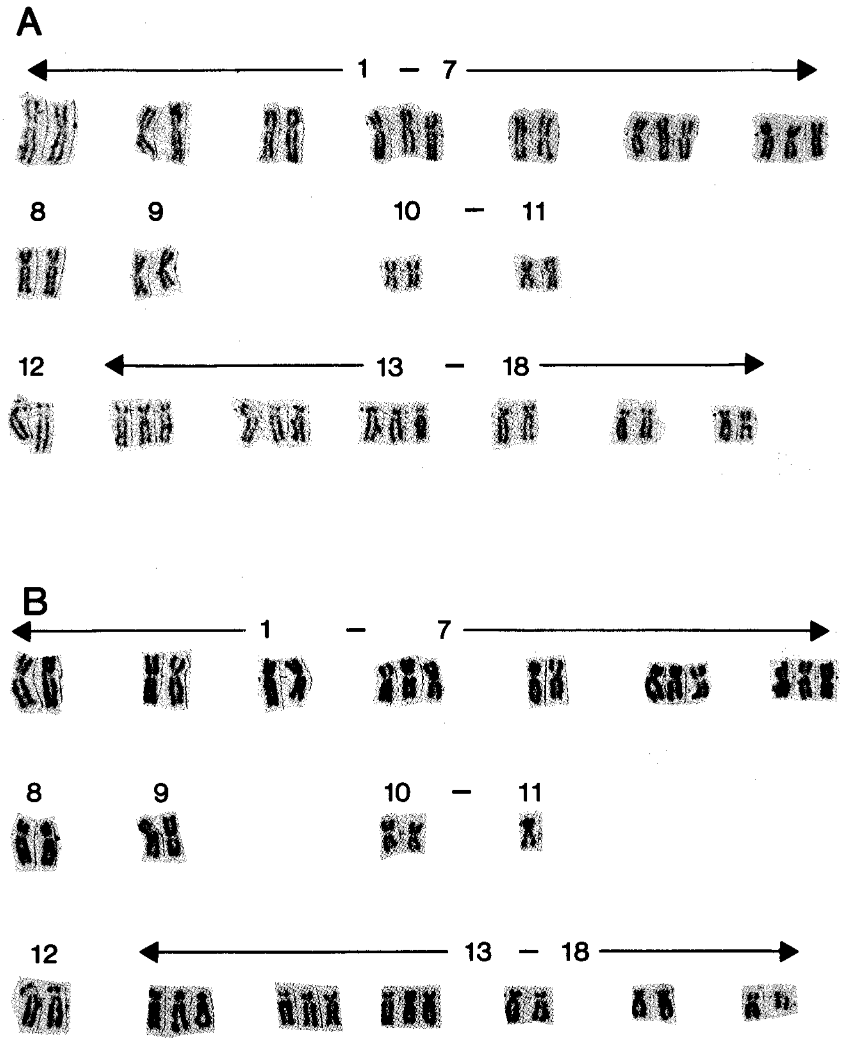

FIGURE 8. Two examples of possible karyotypes from the tumor cell line. Tentative classification of chromosomes according to Tymovska and Fischberg (1973).

poietic cell lineage. Large numbers of granulocytes, including eosinophils, could be obtained from cultures of thymus, spleen, or peritoneal exudates of normal *Xenopus* tadpoles or adults after 2 weeks of culture *in vitro* in medium supplemented with 20% A6 supernatant.

The tumor cell line (which we called MAR1), its subclones B3, B7, and B3D3, and breeding nucleus of the *ff* strain necessary for growth *in vivo* of the tumor will be provided to anyone interested in experimenting with this system.

### MATERIALS AND METHODS

1. *Animals*: The colony of *ff* animals (Du Pasquier and Chardonnens, 1975) was started in our institute in 1972, and the *ff* strain has been bred to homozygosity at the MHC locus by brother–sister mating and gynogenesis over the last 18 years. No precise estimation of the degree of homozygosity has been made, but the animals retain skin allografts for more than 200 days. They are considered to be histocompatible. Transplantations were done subcutaneously. Sodium perchlorate ($KClO_4$, 0.1%) was added to the water at stage 48 (Nieuwkoop and Faber, 1967) in order to block development, according to Buscaglia (1977).

2. *Flow cytometry, immunohistology, and regular histology*: The tumor tissue was embedded in plastic for semithin section and stained with Notch's reagent. Cryosections were done at −20°C after embedding in OCT medium (Miles), and immunohistology was performed, as described (Du Pasquier et al., 1985), with various monoclonal antibodies against B- and T-cell markers and fluorescent second reagents (FabII goat antimouse Silenius FITC). Cells stained with monoclonal antibodies were analyzed by flow cytometry on a FACScan apparatus, using $10^5$ cells per sample, as described (Du Pasquier and Flajnik, 1991).

3. *DNA preparation, Southern blotting*: DNA was extracted from tumor cells by conventional phenol chloroform procedure. To avoid degradation, it was crucial to use 0.1 M EDTA in the lysis buffer and not to exceed a concentration of $3 \times 10^7$ cells/ml. Other methods, such as salting out or acetone extraction, which work well for extracting DNA from red blood cells, gave poor results with the tumor. Southern blotting was done as already described (Schwager et al., 1988), loading 10 $\mu$g of DNA per lane. MHC $\alpha 3$ probe was a kind gift of Martin Flajnik (Flajnik et al., 1990a), $V_H7$ probe was obtained from a cDNA cloned by Melanie Wilson.

The $J_H$ probe of 2 kb was obtained by PCR amplification with primers specific of the 5' and 3' region of the $J_H$ (L. Du Pasquier and M. Courtet, unpublished), (3'=1333: TTTAATAATTTCGTAACTAAAACTGCT, and 5'=1976: GGATATTGCTTGATAGGAAATCTCTGAAAT), and labeled by random priming. Twenty million counts were used for hybridization of filters 20× 20 cm.

4. *Cell culture conditions*. The medium used as a base in all cultures *in vitro* was a modification of the one used previously for *in vitro* antibody production or T-cell proliferation assays (Blomberg et al., 1980). The present formula is Leibovitz L15 (Gibco): 400 ml; tridistilled water: 126 ml; sodium acetate (5.6 M), 15 g/l: 76 ml; glutamine (KC Biological) 200 mM: 10 ml; glucose 5 M 40 g/l: 8 ml; Iscove Modified Dulbecco Eagle Minimal Essential Medium (IMDMEM, Gibco): 80 ml; Penicillin-Streptomycin mixture (Gibco) 1000 $\mu$g/ml: 7 ml; Fetal calf serum (FCS, Amimed or Gibco): 36 ml.

The A6 kidney cell line (Rafferty, 1969) was obtained from the American Tissue Culture Collection and adapted to the same medium. When cells grown in 162 $cm^2$ flasks with 100 ml of medium were at confluence, the supernatant was collected and filtered on Nalgene units (0.22 $\mu$) and used within 2 weeks; 200 ml of supernatant were added to the previously mentioned volume of L15 base.

Single tumor cells or fragments were cultured in 24-well Costar plates. When the stable cell line B3 was established, it was cloned by limiting dilution in 96-well Costar plates. Mass production of the cells was established either in 75 or 16 $cm^2$ bottles or in Nunc cell factories of 6000 $cm^2$ with 1000 ml of medium. All cultures were kept at 27°C in a humidified air–$CO_2$ atmosphere (3% $CO_2$) at pH 7.5. Cells from cultures, wells, or flasks, were collected for flow cytometry analysis, cytospun, and stained with Giemsa (Merck). Cultures were kept for a week before collection. For long-term production, cultures were split every week and fed with fresh medium. Normal tissues (thymus and spleen) were dilacerated directly in the culture well. Half of the medium was replaced every 3 days and cells collected at various time intervals were cytospun and stained with Giemsa.

5. Chromsome preparation was as described (Du Pasquier et al., 1985), from cultured cells where colcemid (DIFCO) was added for 4 h at a final concentration of 0.3 $\mu$g/ml. Hypotonic shock was given for 30 min in a 0.02-M KCl solution.

*(Received March 2, 1992)*

*(Accepted March 2, 1992)*

### ACKNOWLEDGMENTS

We thank Michèle Courtet, Brigit Kugelberg, and C. Guiet for excellent technical assistance, Dr. Ronald Palacios, Melanie Wilson, and Charley Steinberg for suggestions and critical reading of the manuscript, and Janette Millar for typing it.

The Basel Institute for Immunology was founded and is supported by F. Hoffmann-La Roche Ltd., Basel, Switzerland.

### REFERENCES

Asfari M. (1988). Mycobacterium-induced infectious granuloma in *Xenopus*: Histopathology and transmissibility. Cancer Res. **48,** 958–963.

Asfari M., and Thiébaud G.H. (1988). Transplantation studies of a putative lymphosarcoma of *Xenopus*. Cancer Res. **48:** 954–957.

Balls M. (1962). Spontaneous neoplasm in amphibian: A review and description of six new cases. Cancer Res. **22:** 1142–1154.

Balls M., Clothier R., and Knowles K.R. (1981). A transplantable large cell lymphoblastic lymphoma in *Xenopus laevis*. Cell Biol. Int. Rys. **5** (Suppl A): 1.

Balls M., Clothier R., and Knowles K.R. (1983) Tumor Incidence in NMU-treated *Xenopus laevis*. *In*: Proceedings of the International Colloquium on Pathology of Reptiles and Amphibians, Vago C., and Matz G., Eds. (Angers: University Press), pp. 165–172.

Balls M., and Ruben L.N. (1967). The transmission of lymphosarcoma in *Xenopus laevis*, the South African clawed toad. Cancer Res. **27:** 654–659.

Balls M., and Ruben L.N. (1968) Lymphoid tumors in amphibia: a review. Prog. Exp. Tumor Res. **10:** 238–260.

Blomberg B., Bernard C.A.A., and Du Pasquier L. (1980) *In vitro* evidence for T–B lymphocyte collaboration in the clawed toad *Xenopus*. Eur. J. Immunol. **10:** 867–876.

Buscaglia M. (1977). Dissociation des déterminants des développements gonadiques, et somatiques au moment de la métamorphose chez le *Xénopus (Xenopus laevis, Daud)*. CR des Séances SPHN Genève **11:** 53–58.

De Lanney L.E., and Blackler K. (1969). Acceptance and regression of strain-specific lymphosarcoma in Mexican Axolotls. *In*: Biology of amphibian tumors, Mizell M., Ed. (New York: Springer Verlag), pp. 300–408.

De Lanney L.E., Prahlad K.V., and Meier A.H. (1964). A malignant lymphoma in the Mexican axolotl. Amer. Zool. **4:** 279.

Du Pasquier L., Blomberg B., and Bernard C.C.A. (1979). Ontogeny of immunity in amphibians: Changes in antibody repertoire and appearance of adult MHC antigens in *Xenopus*. Eur. J. Immunol. **9:** 900–906.

Du Pasquier L., and Chardonnens X. (1975). Genetic aspects of the tolerance to allografts induced at metamorphosis in the toad *Xenopus laevis*. Immunogenetics **2:** 431–440.

Du Pasquier L., and Flajnik M.F. (1991). Expression of MHC class II antigens during *Xenopus* development. Dev. Immunol. **1:** 85–95.

Du Pasquier L., Flajnik M.F., Guiet C., and Hsu E. (1985). Methods to study the immune system of *Xenopus (Amphibia Anura)*. *In*: Immunology Methods III, Lefkovits I., and Pernis B., Eds. (New York: Academic Press), pp. 425–465.

Duryee W.M. (1969). Dependence of tumor formation in frogs or abnormal nuclear function. *In*: Biology of amphibian tumors, Mizell M., Ed. (New York: Springer Verlag), pp. 82–100.

Flajnik M.F., Canel C., Kramer J., and Kasahara M. (1990a) Evolution of the major histocompatibility complex: Molecular cloning of MHC class I from the amphibian *Xenopus*. Proc. Natl. Acad. Sci. USA **88:** 537–541.

Flajnik M.F., Ferrone S., Cohen N., and Du Pasquier L. (1990b). Evolution of the MHC: Antigenicity and unusual tissue distribution of *Xenopus* (frog) class II molecules. Mol. Immunol. **27:** 451–462.

Flajnik M.F., Hsu E., Kaufman J.F., and Du Pasquier L. (1988). Biochemistry, tissue distribution and ontogeny of surface molecules detected on *Xenopus* hemopoietic cells. *In*: Differentiation antigens on lymphohemopoietic tissues. Miyasaka M., and Trnka Z., Eds. (New York: M. Dekker), pp. 387–419.

Flajnik M.F., Kaufman J., Hsu E., Manes M., Parisot R., and Du Pasquier L. (1986). Major histocompatibility complex-encoded class I molecules are absent in immunologically competent *Xenopus* before metamorphosis. J. Immunol. **137:** 3891–3899.

Flajnik M.F., Taylor E., Canel C., Grossberger D., and Du Pasquier L. (1991). Reagents specific for MHC class I antigens of *Xenopus*. Am. Zool. **31:** 580–590.

Hadji-Azimi I. (1970a). Some characteristics of the "lymphoid tumour" inducing agent of *Xenopus laevis*. Experientia **26:** 895–897.

Hadji-Azimi I. (1970b). Transmission of the "lymphoid tumour" of *Xenopus laevis* by injection of cell-free extracts. Experientia **26:** 894–895.

Haire R., Amemiya C.T., Suzuki D., and Litman G.W. (1990). Eleven distinct $V_H$ gene families and additional patterns of sequence variations suggest a high degree of immunoglobulin gene complexity in a lower vertebrate *Xenopus laevis*. J. Exp. Med. **171:** 1721–1737.

Hsu E., and Du Pasquier L. (1984) Studies in *Xenopus* immunoglobulins using monoclonal antibodies. Mol. Immunol. **21:** 257–270.

Hsu E., Flajnik M.F., and Du Pasquier L. (1985). A third immunoglobulin class in amphibians. J. Immunol. **135:** 1998–2004.

Hsu E., Lefkovits I., Flajnik M., and Du Pasquier L. (1991). Light chain heterogeneity in the amphibian *Xenopus*. Mol. Immunol. **28:** 985–994.

Inoue S., Singer M., and Hutchinson J. (1965). Causative agent of a spontaneous originated visceral tumor in the newt, *Triturus pyrrhogaster*. Nature **205:** 408–409.

Nagata, S. (1985). A cell surface marker of thymus dependent lymphocytes in *Xenopus laevis* is identified by mouse monoclonal antibody. Eur. J. Immunol. **15:** 837–841.

Nieuwkoop P.D., and Faber J. (1967) Normal table of *Xenopus laevis* (Amsterdam: North Holland).

Pflugfelder O. (1961). Weitere Untersuchungen über die thyreostatische Wirkung von Kaliumperchlorate bei Amphibien. Roux'Arch. für Entwicklungmech. **153:** 236–254.

Rafferty K.A. (1969) Mass culture of amphibian cells: Methods and observations concerning stability of cell type. *In*: Biology of amphibian tumors, Mizell M., Ed. (New York: Springer Verlag), pp. 52–81.

Schochet S.S. and Lampert P.W. (1969) Plasmocytoma in a *Rana pipiens*. *In*: Biology of amphibian tumors, Mizell M., Ed. (New York: Springer Verlag), pp. 204–214.

Schwager J., Grossberger D., and Du Pasquier L. (1988). Organization and rearrangement of immunoglobulin M genes in the amphibian *Xenopus*. EMBO J. **7:** 2409–2415.

Tymovska J., and Fischberg M. (1973). Chromosome complements of the genus *Xenopus*. Chromosoma **44:** 335–342.

Weiss N., and Du Pasquier, L. (1973). Factors affecting the reactivity of amphibian lymphocytes in a miniaturized technique of the mixed lymphocyte culture. J. Immunol. Meth. **3:** 273–285.

